# Inspiratory muscle training and cardiac autonomic function in healthy adults: a systematic review of acute and chronic responses

**DOI:** 10.3389/fcvm.2026.1935324

**Published:** 2026-09-04

**Authors:** Fahad Hamoud Algharbi, Shibili Nuhmani, Mohammed Alsubaiei, Alsayed Shanb, Maher Alquaimi, Abdullah Alsulayyim, Abdullah Alzahrani

**Affiliations:** 1 Department of Physiotherapy, College of Applied Medical Sciences, Imam Abdulrahman bin Faisal University, Dammam, Saudi Arabia; 2 Medical Support Services, Royal Commission Health Services Program, Royal Commission for Jubail and Yanbu, Jubail, Saudi Arabia; 3 Department of Respiratory Therapy, College of Applied Medical Sciences, Imam Abdulrahman bin Faisal University, Dammam, Saudi Arabia; 4 Respiratory Therapy Program, Department of Nursing, College of Nursing and Health Sciences, Jazan University, Jazan, Saudi Arabia; 5 General Administration of Quality and Patient Safety, Deputyship of Planning and Transformation, Ministry of Health, Riyadh, Saudi Arabia

**Keywords:** cardiac autonomic function, heart rate variability, inspiratory muscle training, primary prevention, sympathovagal balance, vagal modulation

## Abstract

**Background:**

Reduced heart rate variability (HRV) is an early marker of cardiac autonomic decline and predicts arrhythmia, sudden cardiac death, and heart failure. Inspiratory muscle training (IMT) is a low-cost, self-administered intervention that may modulate autonomic function, but prior reviews have focused on clinical populations or older adults. No synthesis has examined HRV responses to IMT in healthy adults or distinguished acute from chronic effects. This review evaluated the effects of acute and chronic IMT on HRV in healthy adults and explored dose–response relationships.

**Methods:**

Six databases (PubMed, Scopus, Web of Science, MEDLINE, EMBASE, Dimensions) were searched from inception to April 2026. Controlled trials of IMT in healthy adults (≥18 years) reporting autonomic outcomes were included. Reporting followed PRISMA 2020; risk of bias was assessed with RoB 2 and ROBINS-I. Given substantial methodological heterogeneity, evidence was synthesized narratively (PROSPERO CRD420261352310).

**Results:**

Thirteen trials (2013–2024; 10 independent cohorts; 214 participants, aged 18–80 years) were included, comprising six chronic (4–11 weeks) and seven acute reports. IMT modulated HRV in an intensity-, duration-, and population-dependent manner. Low-to-moderate loading (30%–60% MIP) was associated with increases in vagal-related HRV indices; however, these changes may partly reflect alterations in breathing pattern and respiratory mechanics. Changes were detectable from week 2 in chronic studies and were reversed following detraining. Loading above ∼60% MIP activated the inspiratory muscle metaboreflex and was associated with a relative shift toward higher normalized LF and lower HF power. The proximate mechanism appeared to be remodeled breathing pattern and enhanced cardiorespiratory coupling rather than altered baroreflex sensitivity. Certainty of evidence was very low across outcomes, reflecting small samples and a narrow demographic base.

**Conclusions:**

In healthy adults, IMT at 30%–60% MIP was associated with favorable changes in vagal-related HRV indices, although whether these reflect true vagal adaptation or altered respiratory mechanics remains unresolved. A protocol of 50% MIP, 30 breaths, five sessions weekly for ≥4 weeks, with standardized paced breathing during HRV assessment to minimize respiratory confounding, is proposed as a reference condition for future IMT–HRV trials.

**Systematic Review Registration:**

https://www.crd.york.ac.uk/PROSPERO/view/CRD420261352310, PROSPERO (CRD420261352310).

## 1 Introduction

Cardiac autonomic modulation reflects the balance between sympathetic and parasympathetic (vagal) input to the sinoatrial node and is commonly assessed using heart rate variability (HRV), defined as beat-to-beat variation in R–R intervals (1). Reduced HRV is a marker of autonomic dysfunction and is associated with increased risk of arrhythmias, sudden cardiac death, and incident heart failure across age groups (2). HRV is quantified using complementary metrics: time-domain indices (e.g., RMSSD, SDNN, pNN50), frequency-domain indices (high-frequency power, 0.15–0.40 Hz, reflecting vagal modulation; low-frequency power, 0.04–0.15 Hz, reflecting baroreflex and mixed autonomic influences; and the LF/HF ratio), and non-linear indices (e.g., SD1/SD2, sample entropy) that capture signal complexity (3, 4).

Inspiratory muscle training (IMT) is a non-pharmacological intervention involving breathing against an external load, typically prescribed relative to maximal inspiratory pressure (MIP) (5). Two protocols are widely used: moderate-intensity, higher-volume training (30 breaths at 30%–60% MIP, twice daily) and high-resistance, low-volume inspiratory strength training (IMST; 30 breaths at approximately 75% MIP, once daily) (6). Randomized trials in adults with above-normal blood pressure show that IMST reduces resting and ambulatory systolic blood pressure, improves endothelial function, and increases nitric oxide bioavailability, with effects comparable to aerobic exercise (7, 8). Meta-analyses in hypertensive populations report reductions of approximately 8–13 mmHg systolic and 4–5 mmHg diastolic blood pressure (9, 10).

Inspiratory muscle training has been investigated as a modulator of cardiac autonomic function across a range of clinical populations. Controlled trials and a prior systematic review indicate that low-intensity IMT can chronically enhance parasympathetic modulation and attenuate sympathetic modulation, as assessed by spectral analysis of HRV, in patients with essential hypertension, type 2 diabetes mellitus, chronic heart failure, and chronic obstructive pulmonary disease (11, 12). However, prior systematic reviews of IMT and cardiovascular autonomic outcomes have been limited in scope and population. De Abreu et al. (12) synthesized a small number of studies conducted predominantly in patients with established cardiovascular, metabolic, or pulmonary disease; Cipriano et al. (13) pooled heterogeneous trials, most involving participants with established cardiovascular disease; and Manifield et al. (14) focused on older adults and reported primarily functional outcomes, with limited HRV data. No review has synthesized HRV responses to IMT specifically in healthy adults across a broad age range, distinguished acute from chronic effects, or examined dose–response relationships for autonomic outcomes, a gap of direct relevance to primary prevention.

Several mechanisms may link IMT to autonomic modulation. Slow, deep breathing increases tidal volume and prolongs expiration, enhancing respiratory sinus arrhythmia and high-frequency HRV (15). High-frequency power indexes vagal modulation only when respiration remains within approximately 0.15 to 0.40 Hz, and respiratory sinus arrhythmia amplitude is influenced by respiratory rate and tidal volume independently of vagal tone (16). Changes in high-frequency power following a breathing intervention must therefore be interpreted alongside any concurrent change in breathing pattern. Negative intrathoracic pressure during loaded inspiration increases venous return and activates cardiopulmonary receptors, reducing sympathetic outflow (17). At higher intensities, inspiratory muscle fatigue activates a metaboreflex mediated by group III/IV afferents, increasing sympathetic activity and vascular resistance (18–20). Chronic IMT may attenuate this response by improving inspiratory muscle capacity. IMT may also modify breathing patterns and enhance cardiorespiratory coupling, with potential effects on baroreflex function and cerebral autoregulation (21, 22).

Most IMT studies have been conducted in clinical populations with established cardiopulmonary disease, and it remains unclear whether IMT improves autonomic function in healthy adults. This question is central to preventive medicine. Although such adults carry no diagnosis, several large occupational and environmental groups are repeatedly exposed to stressors that erode cardiac autonomic function long before overt disease appears. Shift workers and emergency responders endure chronic circadian disruption together with recurrent extreme physiological and psychological stress, a combination associated with reduced HRV, impaired baroreflex control, orthostatic dysregulation, and elevated long-term cardiovascular risk (23, 24). Individuals treated with neurotoxic chemotherapeutic agents, and those recovering from cardiotropic viral illness, likewise show diminished HRV and autonomic instability (25, 26). In each of these groups the autonomic deficit is acquired, measurable, and potentially reversible, yet none constitutes a clinical cardiovascular population in whom IMT has already been tested. Establishing that IMT enhances cardiac autonomic function in healthy adults would therefore provide the evidentiary basis for deploying it as a low-cost, self-administered, non-pharmacological strategy to protect precisely these higher-risk but non-clinical populations.

This systematic review evaluates the effects of acute and chronic IMT on cardiac autonomic function in healthy adults, with HRV as the primary outcome domain and muscle sympathetic nerve activity, baroreflex sensitivity and cardiorespiratory coupling as secondary autonomic outcomes. It addresses the following questions: which HRV indices are most responsive to acute and chronic IMT; the minimum effective dose in terms of intensity, volume, frequency, and duration; the persistence of HRV changes after training cessation; how acute responses compare with chronic adaptations; whether responses vary by age, sex, or fitness status; which HRV measurement protocols are most appropriate (e.g., recording duration, posture, breathing control, timing); and which intervention and measurement features should be standardized in future randomized trials.

## 2 Materials and methods

This systematic review was conducted and reported in accordance with the Preferred Reporting Items for Systematic Reviews and Meta-Analyses (PRISMA) 2020 statement (27) and was prospectively registered with the International Prospective Register of Systematic Reviews (PROSPERO; registration number CRD420261352310). Because of anticipated methodological heterogeneity across studies, no quantitative meta-analytic pooling was undertaken, and the evidence was synthesized narratively.

### 2.1 Eligibility criteria

Randomized controlled trials (RCTs), non-randomized controlled trials with a comparator group, and randomized crossover studies examining IMT in healthy adults (≥18 years) were included. Eligible participants were free from diagnosed cardiovascular, pulmonary, metabolic, or neurological disease; studies involving clinical populations (e.g., heart failure, coronary artery disease, treated hypertension, chronic obstructive pulmonary disease, asthma, type 2 diabetes, chronic kidney disease, obstructive sleep apnea) were excluded. Interventions included any IMT modality using resistive or pressure-threshold loading devices, delivered at any intensity, frequency, or duration. Studies involving combined inspiratory and expiratory training, slow-breathing interventions without inspiratory loading, or incentive spirometry alone were excluded. Comparators included sham IMT, no intervention, placebo, active control, or alternative IMT intensities; crossover studies were eligible if a washout period was reported. Primary outcomes were HRV indices: time-domain (RMSSD, SDNN, pNN50), frequency-domain (HF, LF, LF/HF, total power) and non-linear (SD1, SD2, sample entropy, symbolic dynamics). Secondary autonomic outcomes were muscle sympathetic nerve activity (MSNA), baroreflex sensitivity (BRS) and cardiorespiratory coupling. Further secondary outcomes were MIP, blood pressure, heart rate, arterial stiffness and respiratory function. Studies were required to report at least one primary or secondary autonomic outcome. Studies were excluded if they were not peer-reviewed or were not published in English. Acute single-session studies in which participants served as their own comparator across loading conditions or against a pre-loading baseline were eligible, consistent with the registered protocol's allowance for within-subject pre–post comparisons.

### 2.2 Information sources and search strategy

A comprehensive search was conducted across six electronic databases (PubMed, Scopus, Web of Science, MEDLINE, EMBASE, and Dimensions) from inception to April 2026. Search strategies were developed in consultation with a medical librarian and combined controlled vocabulary and free-text terms across three concepts: the intervention (“inspiratory muscle training,” “IMT,” “inspiratory muscle strength training,” “IMST,” “respiratory muscle training,” “respiratory muscle strengthening”), the autonomic outcome (“heart rate variability,” “HRV,” “autonomic function,” “autonomic nervous system,” “sympathetic modulation,” “sympathovagal balance,” “muscle sympathetic nerve activity,” “MSNA”), and cardiovascular outcomes (“blood pressure,” “heart rate,” “cardiovascular outcomes”). The complete, database-specific search strings are provided in Supplementary File S1.

### 2.3 Study selection and data extraction

**Figure 1:**
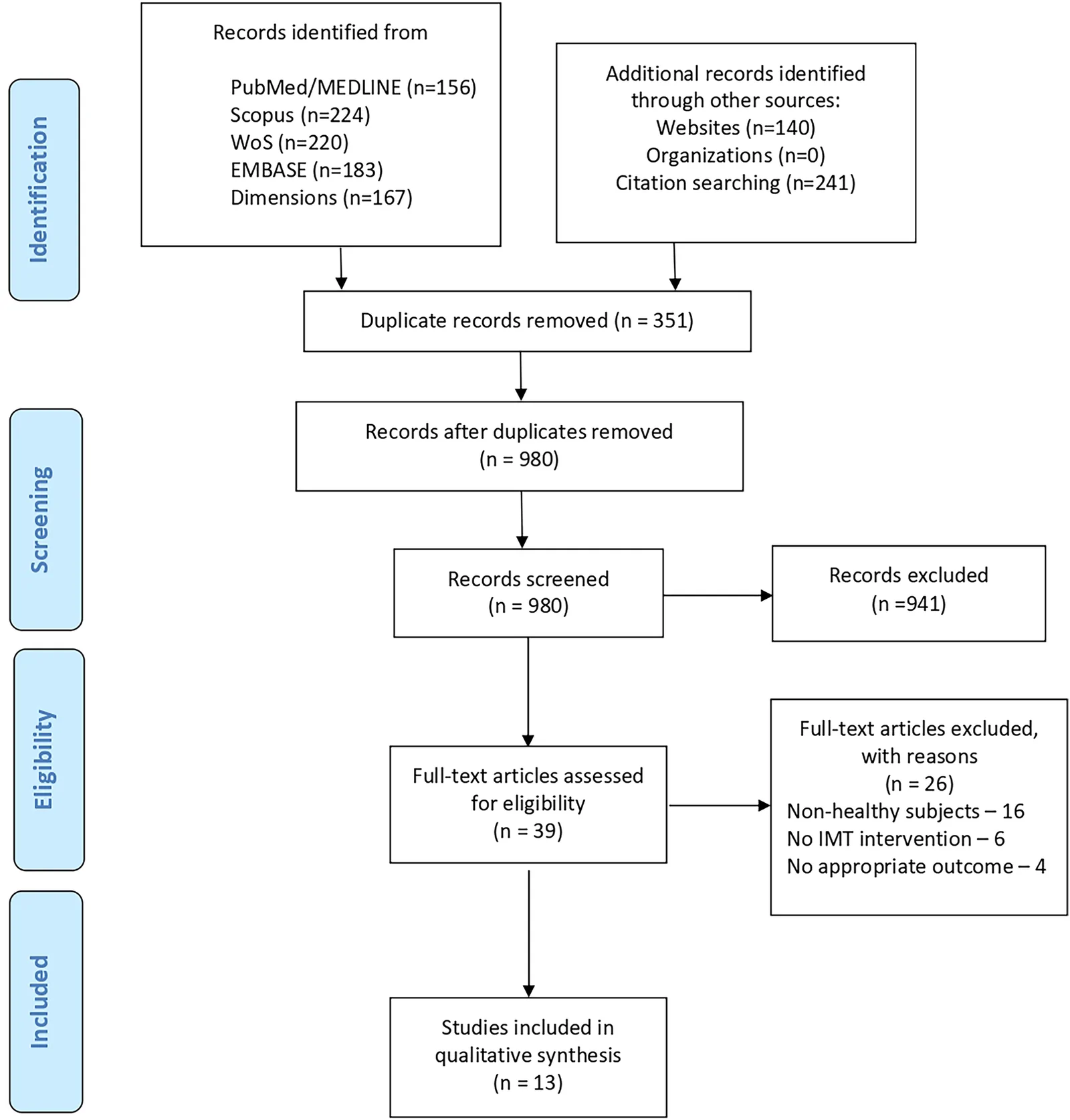
PRISMA flow diagram showing study selection process from initial database selections through final inclusion.

Two reviewers (FHA, ASh) independently screened titles and abstracts against the eligibility criteria, retrieved full texts of potentially eligible studies, and assessed them for inclusion, using Covidence to manage records and conflicts. Disagreements were resolved by consensus or, where required, by consultation with a third reviewer. The study selection process is presented in a PRISMA 2020 flow diagram (Figure 1). Two reviewers independently extracted data using a piloted extraction form capturing study design, population, and IMT protocol (device, intensity, volume, frequency, duration, pacing), HRV recording methodology (device, position, duration, breathing condition), HRV indices, quantitative outcomes with effect sizes, and reported mechanisms. Reports from the same participant sample were identified by comparing authors, sites, enrolment periods and baseline characteristics, and were linked to a single cohort. Participants were counted once, and concordant findings from linked reports were not treated as independent replication.

### 2.4 Risk of bias

**Figure 2:**
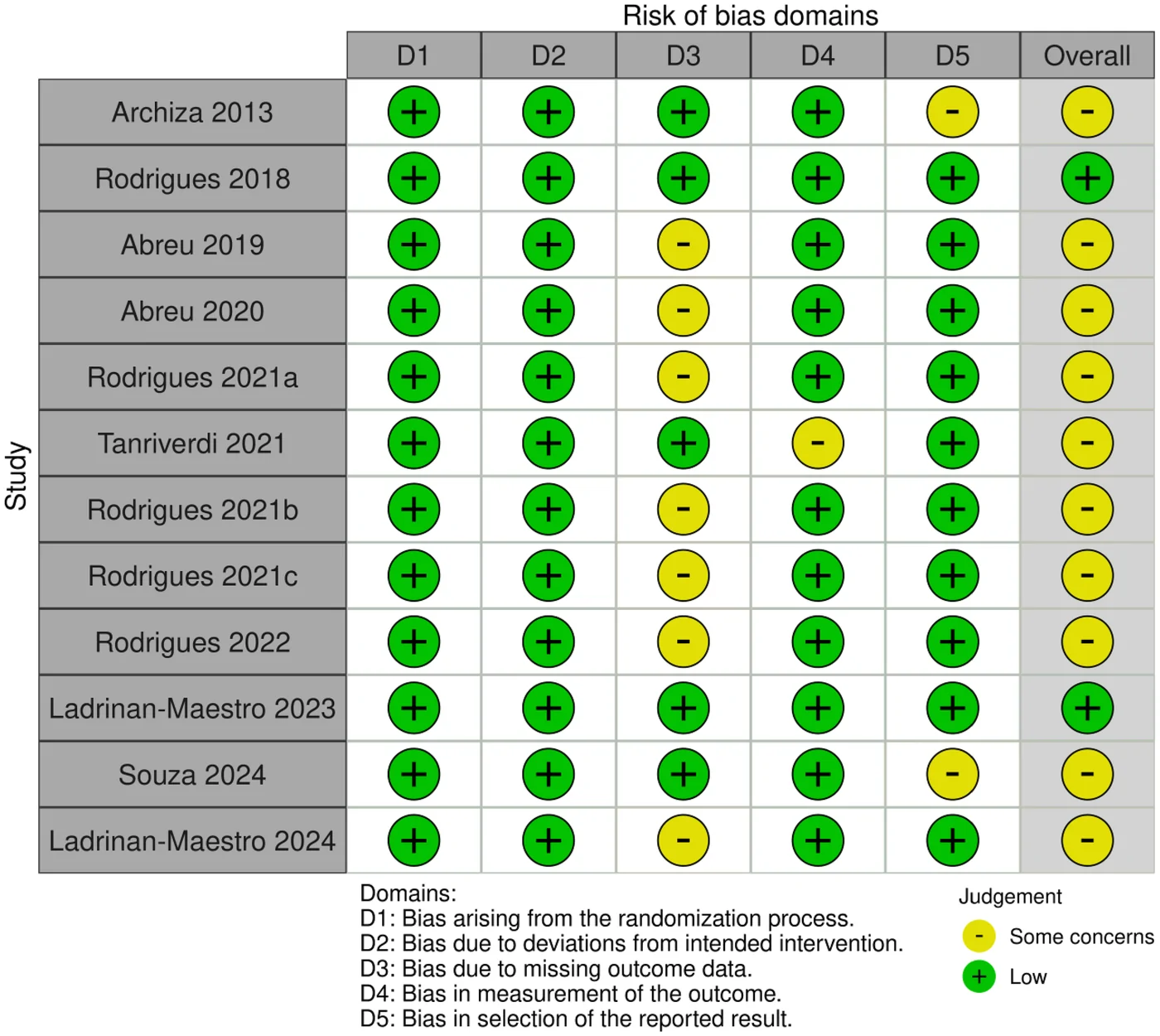
Risk-of-bias traffic-light plot (RoB 2) for the twelve randomised reports, showing domain-level and overall judgements by study, using a colour-blind-safe palette.

Risk of bias in randomized studies was assessed using the Cochrane Risk of Bias 2 (RoB 2) tool across five domains: randomization process, deviations from intended interventions, missing outcome data, measurement of the outcome, and selection of the reported result (28). Non-randomised controlled studies were assessed using the ROBINS-I tool (29). Two reviewers (FHA, ASh) independently performed all assessments, with disagreements resolved by consensus; domain-level and overall judgements for the randomized studies are presented as a traffic-light figure (Figure 2); the single non-randomized study is reported in the text and in Supplementary Table S2.

### 2.5 Data synthesis

Given methodological heterogeneity across studies, including differences in HRV recording protocols (duration, posture, breathing control), IMT intensity, and study design, formal meta-analysis was not considered appropriate, and results were synthesized narratively; study characteristics are shown in Table 1. Findings were structured according to pre-specified subgroups: (1) acute (single-session) versus chronic training designs; (2) IMT intensity strata (≤30%, 30%–60%, ≥60% MIP); (3) age groups (18–35, 35–60, >60 years); and (4) sex. This approach enabled comparison of intervention effects across key methodological and physiological dimensions (30).

**Table 1:** Characteristics of the included studies.

Study (year, country)	Design	Population	N analyzed; sex	Duration	Key findings (effect size/p; direction)
Archiza 2013 (Brazil)	RCT crossover, double-blind	Healthy older men	25; M	Single session	RMSSD, SD1, HF(abs) ↓ at 80% vs. 30% MIP (*p* < 0.05). Direction: ↓ vagal with load.
Rodrigues 2018 (Brazil)	RCT, double-blind, placebo-controlled	Older women	19 (IMT 11/PLA 8); F	5 weeks	HFn ↑ from week 2; LF/HF ↓ from week 1 (HFn ES 1.13→1.43; LF/HF ES 0.83→0.70). Direction: chronic vagal gain.
Abreu 2019 (Brazil)	RCT, blinded	Amateur cyclists, 20–40 y	30 (Sham 9/MIP60 10/CIP 11); M	11 weeks	HF(abs) ↑ at rest in MIP60; CIP unchanged or worse (521→1,161 ms^2^; *p* < 0.05). Direction: HF power ↑ (MIP60); no improvement (CIP).
Abreu 2020 (Brazil)	RCT, transfer-entropy analysis	Same cohort as Abreu 2019	30; M	11 weeks	Conditional transfer entropy (CRC) ↑ in MIP60 under standing (*p* < 0.05). Direction: improved coupling under stress.
DeLucia 2021 (USA)	Acute experimental, microneurography	Healthy young, 18–30 y	14 (7F/7M); both	Single session	MSNA burst frequency ↓ 41% during IMST (95% CI –52.5 to –29.8). Direction: acute sympathetic suppression.
Rodrigues 2021a (Brazil)	Randomised crossover	Healthy older women	10; F	Single session (30 breaths)	HFn ↑, LFn ↓, 2UV ↑ at 30% MIP (ES 1.12, 1.16, 1.14). Direction: acute vagal enhancement.
Tanrıverdi 2021 (Türkiye)	Prospective crossover	Healthy young, 18–25 y	36 (18F/18M); both	Single session × 4	RMSSD, SDNN, pNN50 ↑ at DBE/10/30%; AoPWV ↑ at 60% (*p* < 0.05 vs. DBE). Direction: vagal HRV indices ↑ at DBE, 10% and 30%; AoPWV ↑ without HRV change at 60%.
Rodrigues 2021b (Brazil)	RCT, double-blind	Older women	14 (IMT 8/Sham 6); F	4 weeks	HF ↑, LF ↓, LF/HF ↓ after 4-wk IMT (ES 2.70, 2.13, 0.81). Direction: chronic vagal gain.
Rodrigues 2021 c (Brazil)	RCT, double-blind	Older women (same cohort Rodrigues 2021b)	12 (IMT 6/Sham 6); F	4 weeks + 4 weeks detraining	HFn and MIP gains reversed after 4 weeks without training
Rodrigues 2022 (Brazil)	RCT (cerebrovascular outcomes)	Same cohort as Rodrigues 2021b	14; F	4 weeks	↑ LF oscillations in BP & MCAv; TFA gain ↓ (*p* = 0.01–0.04). Direction: cerebrovascular benefit.
Ladriñán-Maestro 2023 (Spain)	RCT, double-blind, 3 arms	Healthy youth, 18–45 y	27 (3 × 9); both	Single session	Fatigue: LF ↑20%, HF ↓22%; Activation: opposite (ES 1.45–1.95 fatigue; 0.59–1.95 activation). Direction: fatigue = sympathetic; activation = vagal.
Souza 2024 (Brazil)	Randomised crossover	Healthy sedentary young men, 25 ± 5 y	15; M	Single session × 4	RMSSD/pNN50 ↑ during 60-min recovery (all intensities incl. sham) (*η*p^2^ 0.28–0.34). Direction: recovery increase in PPG-derived indices; occurred in sham; not ECG-derived.
Ladriñán-Maestro 2024 (Spain)	RCT, double-blind, 3 arms	Older adults, >60 y	24 (3 × 8); both	Single session	Fatigue: LF ↑, HF ↓, RMSSD ↓; Activation: opposite (ES 0.57–2.80). Direction: fatigue more pronounced than in young.

M, male; F, female; IMT, inspiratory muscle training; PLA, placebo; MIP, maximal inspiratory pressure; MIP60, 60% MIP; CIP, critical inspiratory pressure (∼80%–90% MIP); ES, effect size (Cohen's d unless stated); ηp^2^, partial eta-squared; AoPWV, aortic pulse-wave velocity; MSNA, muscle sympathetic nerve activity; CRC, cardiorespiratory coupling; TFA, transfer-function analysis; MCAv, middle cerebral artery velocity; DBE, diaphragmatic breathing exercise; 2UV, two unlike-variation symbolic index. Abreu 2019/2020 share one cohort; Rodrigues 2021b, 2021c and 2022 share another.

The certainty of the evidence for each principal outcome was rated using the GRADE approach across the domains of risk of bias, inconsistency, indirectness, imprecision and publication bias. Because no estimates were pooled, ratings were applied to the direction and consistency of effect rather than to a summary estimate.

## 3 Results

### 3.1 Study selection and characteristics

The PRISMA 2020 flow diagram (Figure 1) summarizes study identification, screening, and inclusion. Thirteen reports published between 2013 and 2024 met the inclusion criteria, providing data on 214 healthy adult participants (age range 18–80 years). The 13 reports represent 10 independent cohorts: Abreu 2019 (31) and Abreu 2020 (32) share one cohort but reported different outcomes, and Rodrigues 2021b (21), Rodrigues 2021c (33), and Rodrigues 2022 (22) share another. Six reports are chronic training trials (4–11 weeks) and seven are acute or single-session investigations. Three Brazilian research groups (UFSCar, UFF, UFJF) contributed nine of the thirteen studies; two originated from Spain, one from Türkiye, and one from the United States. Sample sizes ranged from 10 to 36. Chronic training studies were dominated by older women (Rodrigues series) and male cyclists (Abreu), whereas acute studies showed a more balanced sex distribution. The POWERbreathe device family was used in 11 of 13 studies; HRV was recorded with Polar heart-rate monitors in five studies, electrocardiography in seven, and finger photoplethysmography in one (Souza 2024). The characteristics of included studies are presented in Table 1, and their IMT protocols and HRV measurement methodology in Table 2.

**Table 2:** Inspiratory muscle training protocol (FITT) and heart rate variability measurement methodology of the included studies.

Study	Device	Intensity (% MIP)	Volume/frequency	IMT pacing	HRV recording (device; position; duration; breathing)	HRV indices
Archiza 2013	POWERbreathe	30/60/80% (3 conditions)	4 min/load; single session	12 br/min	Polar S810i (1,000 Hz); sitting; 5 min SB + 4 min/load; 6 cycles analysed	RMSSD, SDNN; LF, HF, LF/HF; SD1, SD2, ApEn
Rodrigues 2018	POWERbreathe Wellness	50% (PLA 5%)	30 breaths × 2/day; 5 d/wk × 5 wk	Spontaneous	Polar RS800cx; sitting; 10 min (300 beats); spontaneous + DB 6 br/min	R–R mean; HFn, LFn, LF/HF
Abreu 2019/2020	PowerBreathe Ironman K5	Sham 6 cmH₂O; MIP60 60%; CIP 80%–90%	1 h/session; 3×/wk × 11 wk	12 br/min	ECG (BioAmp) + Finometer Pro; supine + standing; 15 min rest + 15 min stand (256 beats)	HP mean, *σ*^2^HP; HFaHP, LFaHP, ratio; Complexity Index, Transfer Entropy
DeLucia 2021	Hans-Rudolph bespoke valve	75% PImax	5 × 6 breaths; single session	12 br/min	Lead-II ECG + ccNexfin + microelectrode; semi-upright; beat-to-beat (15-s bins)	HR (instantaneous); MSNA (primary)
Rodrigues 2021a (acute)	POWERbreathe K-5	30% (Sham 5 cmH₂O)	30 breaths; single session	15 br/min	Polar RS800cx; sitting; 10 min (300 beats)	R–R mean; HFn, LFn, LF/HF; symbolic dynamics (0 V,1 V,2UV,2LV)
Tanrıverdi 2021	POWERbreathe K5	DBE/10/30/60%	15 min/session; single session×4	12 br/min	SphygmoCor CvMS (5-min ECG); supine; 5 min; spontaneous	RMSSD, SDNN, pNN50, meanRR; LF, HF, LF/HF, TP
Rodrigues 2021b (chronic)	POWERbreathe Wellness	50% (Sham 5%)	30 breaths ×2/day; 5 d/wk × 4 wk	Spontaneous	ECG + Finometer Pro; supine; 10 min (300 beats); spontaneous	R–R mean, var; HFn, LFn, LF/HF; BRS sequence
Rodrigues 2021c (chronic)	POWERbreathe Wellness	50% (Sham 5%)	30 breaths×2/day; 5 d/wk × 4 wk, then 4 wk detraining	Spontaneous	ECG (Finapres Medical Systems), 1 kHz; sitting; 300 beats; spontaneous	HFn, LFn, LF/HF; VLF, LF, HF absolute
Rodrigues 2022	POWERbreathe Wellness	50% (Sham 5%)	30 breaths ×2/day; 7 d/wk × 4 wk	Spont. + DB 0.1 Hz	ECG + Finometer Pro + TCD; supine; 5 min FB + 5 min DB	R–R LF power, LF MBP, LF MCAv, TFA gain
Ladriñán-Maestro 2023	Big Breathe	60% (fatigue)/40% (activation)	Fatigue to failure/2 × 30; single session	Not specified	Polar H10; supine; 3 min (Kubios HRV 3.1.0); spontaneous	RMSSD, SDNN; LFn, HFn, LF/HF
Souza 2024 (PPG)	POWERbreathe	Sham/30/40/60%	8 × 2 min (30/40%); 4 × 2 min (60%); single session ×4	12–15 br/min	FinometerPro PPG; seated; 10 min baseline + 60-min recovery	SDNN, RMSSD, pNN50; LF, HF, LF/HF, TP
Ladriñán-Maestro 2024	Big Breathe	60% (fatigue)/40% (activation)	As 2023; single session	Not specified	Polar H10; supine; 5 min (Kubios HRV 3.1.0); spontaneous	RMSSD, SDNN; LFn, HFn, LF/HF

MIP, maximal inspiratory pressure; PImax, MIP; DBE, diaphragmatic breathing exercise (unloaded); DB, deep-breathing test; SB, spontaneous breathing; FB, free breathing; HP, heart period; AR, autoregressive spectral analysis; TP, total power; BRS, baroreflex sensitivity; TFA, transfer-function analysis; MBP, mean blood pressure; MCAv, middle cerebral artery velocity; TCD, transcranial Doppler; ApEn, approximate entropy; PPG, finger photoplethysmography. Abreu 2019/2020 share one cohort; Rodrigues 2021b/2022 share another. Interbeat intervals were derived by PPG in Souza 2024 only; all others used ECG or a Polar HR monitor.

### 3.2 Synthesis of HRV outcomes by domain

#### 3.2.1 Time-domain HRV

RMSSD, the most reliable short-term index of cardiac vagal modulation, was reported in five studies, all of which used acute protocols, and showed the clearest intensity-dependent response. Archiza 2013 (34) demonstrated a graded reduction in RMSSD during acute loading, decreasing from 25 ± 3 ms at 30% MIP to 22 ± 2 ms at 60% MIP and 19 ± 2 ms at 80% MIP (*p* < 0.05 vs. 30%), in healthy older men, indicating that even within an acute single-session protocol, higher loads attenuate vagal modulation. The mirror image is provided by the activation arms of the Ladriñán-Maestro studies, in which 40% MIP loading increased RMSSD post-session in both young and older participants (effect size 1.23 in older adults). MSSD was not reported by any chronic report, so no comparison between time-domain and frequency-domain responsiveness to training is possible from these data.

#### 3.2.2 Frequency-domain HRV

Normalized high-frequency power (HFn) was the most frequently reported index to change with chronic IMT; however, no included study directly compared HRV indices to establish greater sensitivity. The Rodrigues series (21, 35) demonstrates the most complete time course: HFn increased significantly from week 2 of training at 50% MIP (effect size 1.13), continued to rise through weeks 3–4 (ES 1.43), and was reversed after a period of detraining (33). The LF/HF ratio decreased even earlier, from week 1 of training, suggesting that the sympathovagal balance shifts before frank vagal-power increases are detectable. In cyclists trained for 11 weeks at 60% MIP (Abreu 2019 31), absolute HF power more than doubled, increasing from 521 ± 448 to 1161 ± 879 ms^2^ (*p* < 0.05), at supine rest, while the high-intensity (CIP, 80%–90% MIP) arm showed no improvement or modest deterioration on standing, a critical finding for athletic populations targeting autonomic adaptation.

#### 3.2.3 Non-linear and advanced markers

Five studies employed non-linear analyses. Poincaré-plot SD1, mathematically equivalent to RMSSD/√2, paralleled RMSSD across loads (Archiza 2013 (34). Symbolic dynamics revealed that the 2UV index (a marker of vagally mediated complexity) increased acutely under 30% MIP loading in older women [Rodrigues 2021a (36), ES 1.14]. The Abreu/Porta group (31, 32) provided unique insights via complexity-index and transfer-entropy approaches: 11 weeks of 60% MIP training increased cardiorespiratory coupling specifically during sympathetic stress (standing), revealing an adaptation invisible to spectral analysis at rest (Abreu 2019/2020 (31, 32). DeLucia 2021 (17) used MSNA microneurography, the gold-standard direct measure of sympathetic outflow, to show that acute IMST at 75% MIP suppresses MSNA burst frequency by 41% during loading, with sex-specific recovery (women showed prolonged suppression for 3–5 min post-session).

### 3.3 Acute versus chronic responses

Acute and chronic designs differed systematically in the direction and mechanism of their autonomic effects, and could not be pooled without subgroup separation. In the acute setting, low-intensity loading (∼30% MIP) was associated with increases in ECG-derived vagal-related HRV indices in some studies. In Souza 2024, however, RMSSD and pNN50 were derived from finger photoplethysmography and increased during the 60-minute recovery period across all conditions, including sham. These findings therefore represent pulse rate variability rather than ECG-derived HRV and should not be interpreted as definitive evidence of post-exercise vagal rebound. As derived by PPG, these estimates were not quantitatively compared with ECG estimates; excluding this study did not alter acute or chronic conclusions, as it contributed only to post-session recovery. After several weeks of training, repeated training drove adaptation: vagal indices (principally HFn) and the LF/HF ratio shifted progressively, becoming detectable from week 2 of training, with the predominant mechanism being remodelling of breathing pattern and enhanced cardiorespiratory coupling rather than a primary change in baroreflex sensitivity. The optimal intensity differed by setting and population: approximately 30% MIP for acute vagal gain and 50% MIP for chronic adaptation in older adults (60% MIP in trained cyclists), and a ceiling effect was apparent in both: sustained loading at ≥60% MIP activated the inspiratory muscle metaboreflex acutely, and chronic training at 80%–90% MIP (CIP) over 11 weeks failed to improve, or modestly worsened, autonomic indices. The integrated dose–response relationship is summarized in Table 3.

**Table 3:** Dose–response matrix for inspiratory muscle training and cardiac autonomic outcomes (intensity×duration×frequency).

Intensity (% MIP)	Acute response	Chronic 4-wk response	Chronic 5–11-wk response	Assessment for autonomic targeting
≤10% (sham/placebo)	Minimal acute autonomic change during loading; RMSSD/pNN50 ↑ during recovery across all conditions, including sham (Souza 2024; see footnote)	No measurable HRV change (Rodrigues 2018/2021b placebo)	No measurable HRV change	Not effective
10%–30%	Acute HFn ↑ (Rodrigues 2021a); acute RMSSD ↑ (Tanrıverdi 2021)	Likely effective if used as primary intensity (no dedicated trial)	No dedicated trial	Effective; suitable for vulnerable populations
30%–60%	Acute vagal enhancement (Souza 2024, 30/40%)	Effective at 50% MIP (Rodrigues 2021b/2022; ES 0.81–2.70)	Effective at 50% MIP (Rodrigues 2018) and 60% MIP (Abreu 2019)	Recommended range
60%–75%	Mixed: 60%×15 min ↑ AoPWV (transient) and acute fatigue → sympathetic (Ladriñán 2023/2024); 75% IMST → acute MSNA suppression (DeLucia 2021)	Not formally studied at 4 wk	75%×6 wk: BP and endothelial benefits (Craighead 2021); HRV not a primary outcome	Acute use only; chronic autonomic benefit not established
≥80% (CIP)	Acute sympathetic dominance (Archiza 2013, 80% load)	Not formally studied	11 wk CIP: autonomic outcomes flat or worse (Abreu 2019)	Not supported for autonomic targeting

Intensity range most consistently associated with favourable autonomic outcomes across studies: 30%–60% MIP. The 60% MIP boundary is approximately where sustained loading begins to activate the inspiratory muscle metaboreflex and shift the HRV pattern toward relatively higher LF and lower HF power. MIP, maximal inspiratory pressure; CIP, critical inspiratory pressure; AoPWV, aortic pulse-wave velocity; MSNA, muscle sympathetic nerve activity; BP, blood pressure. Rodrigues 2021a is an acute single-session study; Rodrigues 2021b is a four-week training trial. Sham was not autonomically inert in Souza 2024: RMSSD/pNN50 ↑ during 60-min recovery across all conditions, including sham, suggesting a paced-breathing rather than load effect.

### 3.4 Risk of bias

Twelve of the thirteen included reports used randomized parallel-group or randomized crossover designs and were appraised with RoB 2. Two were rated at low risk of bias overall (Rodrigues 2018 (35); Ladriñán-Maestro 2023 (37) and ten as raising some concerns; none was rated at high risk. Judgements were low risk in every report for the randomization process and for deviations from intended interventions. The most frequent limitation was in the domain covering missing outcome data, which raised some concerns in seven reports (Abreu 2019 (31); Abreu 2020 (32); Rodrigues 2021a (36); Rodrigues 2021b (21); Rodrigues 2021c (33); Rodrigues 2022 (22); Ladriñán-Maestro 2024 (38). Some concerns were raised for measurement of the outcome in one report (Tanrıverdi 2021 (39) and for selection of the reported result in two (Archiza 2013 (34); Souza 2024 (40). Domain-level judgements for these twelve reports are shown in Figure 2.

One study (DeLucia 2021 (17) used a single-arm, non-randomized before-after design and was appraised with ROBINS-I. It was rated at serious risk of bias overall, driven by confounding, with moderate concerns for missing data, measurement of the outcome and selection of the reported result, and low risk for participant selection, classification of the intervention and deviations from intended interventions. Domain-level judgements for this study are reported in Supplementary Table S2. Taken together, the most consistent evidence indicates chronic vagal modulation associated with 50% MIP training in older women and acute vagal modulation with low-intensity (∼30% MIP) loading, while the sympathetic shift produced by sustained loading at or above 60% MIP is reproducible across both young and older participants. All estimates derive from small samples drawn from a limited number of research groups and should be interpreted with corresponding caution.

### 3.5 Minimum effective dose and persistence of effects

Across the included studies, the lowest intensity associated with a detectable autonomic response was 30% MIP for acute vagal modulation (Rodrigues 2021a (36); Tanrıverdi 2021 (39), whereas chronic autonomic improvements were obtained consistently at 50% MIP (Rodrigues 2018 (35); Rodrigues 2021b (21). On this basis a protocol can be proposed for future trials: an intensity of approximately 30% MIP for acute effects and 50% MIP for training; a volume of 30 breaths per session; a frequency of twice daily on 5–7 days per week; and a duration of at least 4 weeks, with vagal gains becoming detectable from the second week. This combination, applied almost uniformly across the chronic trials, is the most frequently replicated protocol associated with improvements in vagal-mediated HRV and reductions in sympathovagal balance. Intensities below 30% MIP produce smaller acute effects, while sustained loading above 60% MIP risks activating the inspiratory muscle metaboreflex and is therefore not recommended for autonomic targeting.

Evidence on the persistence of these adaptations after training cessation comes from a single longitudinal detraining study in twelve older women (33). After four weeks without training, the gains in maximal inspiratory pressure and in vagal-mediated HRV (normalised high-frequency power) were reversed, with autonomic indices returning to baseline values. Based on these limited data, detraining appears to reverse HRV gains, but this requires confirmation in larger cohorts, and retention beyond four weeks of cessation remains uncharacterized.

### 3.6 Certainty of the evidence

Certainty was rated separately for chronic and acute evidence (Table 4). For chronic inspiratory muscle training, certainty was very low for all cardiac autonomic outcomes due to risk of bias (no allocation concealment or intention-to-treat analysis, high attrition), indirectness (older women from one laboratory vs. young male athletes), imprecision (small samples), suspected publication bias, and inconsistency. Certainty for maximal inspiratory pressure was low, given large, consistent effects. For acute inspiratory loading, certainty was also very low, driven primarily by inconsistency: responses varied with load intensity and task failure, and protocols differed in load, fatigue, and timing, preventing clear attribution. One trial showed similar HRV changes after an unloaded sham session, further limiting causal inference. Population indirectness and publication bias were less concerning, as acute trials included diverse cohorts from multiple countries with reported null findings. Time-domain HRV indices were not reported in chronic trials and could not be rated. Muscle sympathetic nerve activity was assessed in one uncontrolled study and rated very low.

**Table 4:** GRADE summary of the certainty of evidence for chronic and acute inspiratory muscle training.

Outcome	Trials (participants)	Risk of bias	Inconsistency	Indirectness	Imprecision	Publication bias	Certainty
Chronic IMT (4 to 11 weeks)*							
Vagal-mediated HRV (HF, HFn, HFa)	4 (75)	Serious^a^	Serious^b^	Serious ^c^	Very serious^d^	Serious ^e^	⨁◯◯◯ Very low
Sympathovagal balance (LF/HF)	4 (75)	Serious^a^	Serious^f^	Serious ^c^	Very serious^d^	Serious ^e^	⨁◯◯◯ Very low
Spontaneous baroreflex sensitivity	3 (74)	Serious^a^	Not serious^g^	Serious ^c^	Very serious^d^	Not serious	⨁◯◯◯ Very low
Cardiorespiratory coupling (transfer entropy, squared coherence)	1 (30)	Serious^h^	Not assessable	Serious^i^	Very serious^d^	Not serious	⨁◯◯◯ Very low
Blood pressure variability (SBP-LF, LFa-SAP)	2 (44)	Serious^a^	Serious^j^	Serious^c^	Very serious^d^	Serious ^e^	⨁◯◯◯ Very low
Maximal inspiratory pressure (fidelity)	4 (75)	Serious^a^	Not serious	Serious ^c^	Not serious^k^	Not serious	⨁⨁◯◯ Low
Acute IMT (Single Session) *							
Vagal-mediated HRV, frequency domain (HF, HFn)	5 (119)	Serious^l^	Very serious	Not serious	Serious	Not serious	⨁◯◯◯ Very low
Vagal-mediated HRV, time domain (RMSSD, pNN50, SDNN)	4 (88)	Serious^l^	Serious	Serious	Serious	Not serious	⨁◯◯◯ Very low
Sympathovagal balance (LF/HF)	5 (119)	Serious^l^	Very serious	Not serious	Serious	Not serious	⨁◯◯◯ Very low
Non-linear HRV (symbolic dynamics, SD1/SD2, ApEn)	2 (35)	Serious^l^	Not assessable	Serious	Very serious^d^	Serious	⨁◯◯◯ Very low
Load-intensity gradient in the acute vagal response	4 (97)	Serious^l^	Serious^s^	Not serious	Serious	Not serious	⨁◯◯◯ Very low
Muscle sympathetic nerve activity	1 (14)	Very serious	Not assessable	Serious	Very serious^d^	Not serious	⨁◯◯◯ Very low

*Contributing trials: Chronic IMT: Rodrigues 2018; Rodrigues 2021b, 2021c and 2022; Martins de Abreu 2019/Abreu 2020. Acute IMT: Archiza 2013; Rodrigues 2021a; Tanrıverdi 2021; Souza 2024; Ladriñán-Maestro 2024 (older adults); Ladriñán-Maestro 2024 (youths); DeLucia 2021.

a Allocation concealment was not reported; no intention-to-treat analysis was performed.

b Sample size was below the optimal information size.

c Participants were predominantly older women from a single laboratory; sex and age were confounded with centre.

d Response direction differed by load intensity and fatigue.

e Registration, allocation concealment, and blinding were inconsistent.

f Small, unregistered trials reported significant findings; publication bias was suspected.

g Protocols were not comparable.

h Effect direction was not consistent across trials.

i Evidence was based on a single trial in a narrow population.

j Uncontrolled design or no formal between-group comparison.

k Controlled breathing alone reproduced the response in the sham condition.

l Mixed across the acute body.

## 4 Discussion

### 4.1 Summary of principal findings

This systematic review of 13 reports demonstrates that IMT modulates cardiac autonomic function in healthy adults in an intensity-, duration-, and population-dependent manner. The evidence supports three consistent observations. First, low-to-moderate-intensity loading (30%–60% MIP) was associated with cardiac vagal modulation across age strata, with acute effect sizes of approximately 1.0–1.2 and chronic effect sizes (for HFn after 4–5 weeks) of 1.1–2.7. Second, sustained or fatiguing loading above approximately 60% MIP triggers the inspiratory muscle metaboreflex and was associated with a relative shift toward higher LF power, a pattern observed in both young and older participants and with the foundational mechanistic literature on group III/IV afferent feedback (18, 20). Third, the proximate mechanism of chronic adaptation appears to be modification of breathing pattern (shorter inspiratory time, larger tidal volume during paced breathing) and enhanced cardiorespiratory coupling, rather than a primary change in baroreflex sensitivity, a refinement of the field's conventional understanding (21, 22).

### 4.2 Physiological interpretation

The intensity-dependent biphasic response observed across the included studies is physiologically plausible. At low loads, slow deep inspiratory efforts increase tidal-volume excursions, prolong the expiratory phase, and augment respiratory sinus arrhythmia, directly increasing high-frequency HRV power. Increased negative intrathoracic pressure during inspiration also augments venous return, stretches the right atrium, and stimulates cardiopulmonary low-pressure receptors that inhibit central sympathetic outflow. The DeLucia 2021 (17) microneurography data quantify this latter effect with unique precision: at 75% MIP, a load capable of generating intrathoracic pressure fluctuations of approximately −60 mmHg, MSNA burst frequency was suppressed by approximately 41% during loading. Critically, this sympathetic suppression occurred without changes in arterial blood pressure, ruling out a baroreflex-mediated mechanism and pointing instead to cardiopulmonary receptor activation.

At higher sustained loads, a competing mechanism dominates: metabolite accumulation in fatiguing inspiratory muscles activates group III/IV afferents, eliciting reflex sympathetic activation, vasoconstriction, and tachycardia, the inspiratory muscle metaboreflex. The Ladriñán-Maestro 2023 (37) and 2024 (38) studies provide the cleanest experimental demonstration of this transition in healthy participants: a fatigue protocol at 60% MIP carried to task failure produced large effect sizes for LF increase (ES 1.45 in young; 0.71 in older) and HF decrease (ES 1.60 in young; 2.80 in older). The magnitude of metaboreflex activation was more pronounced in older adults, consistent with age-related sarcopenia and increased thoracic stiffness that compromise diaphragm endurance. Tanrıverdi 2021 (39) provides complementary evidence: 15 min of 60% MIP loading significantly increased aortic pulse-wave velocity (a marker of vascular sympathetic tone) without HRV gain, suggesting that even acute exposure at this intensity is not autonomically benign.

This mechanistic interpretation requires a methodological caveat. Because respiratory sinus arrhythmia amplitude, and therefore high-frequency power, is modulated by respiratory rate and tidal volume independently of cardiac vagal tone (16), and because inspiratory muscle training is expected to shorten inspiratory time and increase tidal volume during paced breathing, part of the high-frequency and normalized high-frequency gains reported across the included studies may reflect altered respiratory mechanics rather than a primary increase in vagal outflow. This does not negate the autonomic benefit, but it reinforces the need to record and report breathing rate alongside HRV and supports the use of paced breathing to standardize respiratory contributions to high-frequency power. A related caution applies to the LF/HF ratio: low-frequency power is not a specific marker of cardiac sympathetic activity, and the ratio does not reliably quantify sympathovagal balance outside tightly controlled short-term recordings (41). The reductions in LF/HF observed in the chronic trials are therefore most defensibly described as a shift in the spectral distribution of HRV accompanying training rather than as a quantified change in sympathetic tone.

These findings are consistent with an intensity-dependent pattern: above 30% approximately MIP, vagal modulation is evident, whereas above approximately 60% MIP the metaboreflex shifts the balance toward sympathetic activation. The 50% MIP intensity used in the Rodrigues chronic-training series sits squarely within this window, and the consistency of HFn gain in those studies (ES 1.13–2.70) suggests this dose–frequency combination is near-optimal for older healthy adults. The 60% MIP intensity used in Abreu's cyclist trial (31) achieves similar gains in trained men, consistent with the principle that higher relative loads can be tolerated by individuals whose absolute MIP is higher.

### 4.3 Comparison with clinical-population evidence

The healthy-adult findings synthesized here are broadly consistent with the wider literature examining inspiratory muscle training (IMT) in clinical populations characterized by autonomic dysfunction. In patients with hypertension, recent meta-analyses have reported pooled reductions in systolic blood pressure of 7.9–12.6 mmHg and diastolic blood pressure of 3.8–4.8 mmHg, accompanied by modest reductions in LF/HF and increases in HF power, indicating a shift toward greater parasympathetic predominance (9, 10). These findings closely mirror the chronic vagal modulation and reductions in sympathovagal balance observed in the Rodrigues studies included in the present review.

Evidence from other chronic disease populations further supports the autonomic effects of IMT. Studies in patients with chronic heart failure have reported reductions in sympathetic nervous activity alongside improvements in inspiratory muscle strength and functional status (5). Likewise, improvements in autonomic regulation have been reported in individuals with diabetic autonomic neuropathy following IMT (42). In pulmonary disease populations, IMT has been associated with enhanced autonomic modulation and improved exercise tolerance in patients with chronic obstructive pulmonary disease (COPD) (43). Similar observations have also been reported in Parkinson's disease, where respiratory muscle training improved both pulmonary function and cardiovascular autonomic control (44).

Although the magnitude of benefit varies across populations, the direction of change is remarkably consistent. Across cardiovascular, metabolic, pulmonary, and neurological disorders, IMT is generally associated with enhanced parasympathetic modulation and/or reduced sympathetic influence. The convergence of these findings strengthens the biological plausibility of the autonomic adaptations observed in healthy adults and suggests that respiratory muscle conditioning may influence central and peripheral mechanisms involved in autonomic regulation.

The high-resistance inspiratory muscle strength training (IMST) protocol developed by Craighead and colleagues (75% MIP, 30 breaths/day, 6 days/week, 6 weeks) provides an additional perspective. In adults with above-normal systolic blood pressure, this protocol produced substantial improvements in resting and ambulatory blood pressure, endothelial function, nitric oxide bioavailability, and cerebrovascular reactivity (7, 8, 45). However, HRV was not a primary outcome in these studies, and direct evidence regarding autonomic adaptation remains limited. Consequently, whether high-intensity IMST enhances or impairs HRV in normotensive healthy populations remains uncertain. This question is particularly important because several acute studies included in the present review suggest that sustained loading at or above approximately 60% MIP may activate the inspiratory muscle metaboreflex and promote a relative shift toward higher LF power, whereas chronic studies employing moderate intensities (50%–60% MIP) consistently demonstrate favourable autonomic adaptations. Future trials directly comparing moderate- and high-intensity IMT protocols using standardized HRV outcomes are therefore warranted.

### 4.4 Directions for preventive medicine and athletic research

In athletic populations, the Abreu 2019/2020 (31, 32) data provide an instructive caution. Eleven weeks of 60% MIP training in male cyclists improved both resting vagal HRV indices and cardiorespiratory coupling under orthostatic stress, while the parallel CIP arm trained at 80%–90% MIP showed no benefit and a small unfavourable trend in some indices. Even in well-trained athletes, the autonomic ceiling for productive chronic IMT appears to be at or below 60% MIP, consistent with the broader respiratory-muscle-training literature in athletes (46). For preventive applications in non-clinical adults, the 30%–60% MIP range with conventional volume (30 breaths) and frequency (5–7 days per week) appears optimal. The practical profile of IMT is well matched to primary prevention: the time burden is roughly five minutes per session, comparable to time-efficient resistance-training paradigms; the equipment cost is low and incurred only once; and training can be performed seated or supine, indoors, and independently of weather or facility access. These features materially lower the adherence barrier in precisely the higher-risk but non-clinical groups identified at the outset of this review. Shift workers and emergency responders, who sustain chronic circadian disruption and recurrent extreme physiological and psychological stress, and in whom adherence to conventional exercise is poor, are obvious candidates for a brief, self-administered, vagal-enhancing intervention (23, 24, 47). They would therefore be informative populations in which to test IMT, although no included study examined them. The same rationale extends to individuals whose autonomic function has been eroded by neurotoxic chemotherapeutic agents or by cardiotropic viral illness, in whom even partial restoration of vagal modulation may carry prognostic value (25, 26). Whether IMT alters autonomic function in these groups is untested and remains a hypothesis for future trials. In each of these settings IMT is proposed not as a treatment for established disease but as a low-cost preventive measure applied before autonomic dysregulation translates into orthostatic intolerance or other clinical consequences.

### 4.5 Recommendations for HRV measurement methodology

The methodological heterogeneity observed across the included studies underscores the need for standardization and motivates the following defaults for IMT–HRV trials, consistent with the ESC/NASPE Task Force standards and the empirical patterns synthesized here (1, 3). The Task Force recommends a 5-minute stationary recording for short-term analysis (1). For IMT trials, we tentatively suggest a 10-minute recording with the final 5 min analyzed, following a stabilization period (48). Supine is preferred for resting recordings; standing recordings provide complementary information about sympathetic responsiveness but are optional. Paced breathing at 12–15 breaths/min should be regarded as mandatory in both acute and chronic IMT–HRV trials, since high-frequency power is directly modulated by respiratory rate and tidal volume and IMT is expected to alter both; spontaneous-breathing recordings cannot separate autonomic adaptation from respiratory mechanical change. Validated HRV analysis software should be used, and reporting should explicitly state the artefact-correction percentage, the breathing-rate distribution, and the analysis method (FFT, autoregressive, or non-linear) (30). A practical minimum reporting set is RMSSD and absolute HF power for acute studies, and HFn and the LF/HF ratio for chronic studies, with SD1 as a non-linear corroborator of vagal modulation.

### 4.6 Strengths and limitations

This review has several strengths. It is the first synthesis to target healthy adults with the explicit aim of informing primary prevention, whereas prior reviews were either disease-focused or restricted to older adults. It applies contemporary methodology (reporting in accordance with PRISMA 2020, with risk of bias assessed using RoB 2 for randomized studies and ROBINS-I for non-randomized controlled studies), a clear advance over the PEDro-only appraisals of earlier reviews. It integrates acute and chronic designs explicitly, allowing the time-course of autonomic adaptation to be characterized, and it preserves the distinction between the moderate-intensity (Rodrigues-style) and high-intensity (Craighead-style) IMT paradigms, which yield different autonomic profiles. The included studies used validated devices and analysis software and reported effect sizes that support critical appraisal.

The limitations should be weighed against these strengths. The number of included studies is small (*n* = 12), reflecting the novelty of the field, and sample sizes were modest (10–36 participants), limiting precision and raising the possibility of effect-size inflation. The evidence base is demographically and geographically concentrated. Nine of the thirteen reports originate from three Brazilian research groups, and the remainder from Spain, Türkiye and the United States, so the findings may reflect the populations, laboratory practices and measurement conventions of a small number of centers rather than a generalizable response. The evidence is further skewed toward older women (the chronic Rodrigues series) and male cyclists (Abreu), sex-stratified analyses are largely absent, and mid-life adults (35–60 years) are essentially unstudied for autonomic outcomes. Methodological heterogeneity in HRV recording (3–15-minute durations; sitting, supine, or standing; spontaneous versus paced breathing) precluded quantitative pooling and limits cross-study comparability. A further source of heterogeneity is the signal from which variability was derived. Whereas most studies used electrocardiography or a bioamplifier, one recorded variability from finger photoplethysmography. Pulse rate variability is increasingly recognized as a distinct biomarker rather than a surrogate for HRV, since the transformation from the electrocardiographic R wave to the photoplethysmographic pulse introduces variability from the pre-ejection period and pulse transit time, producing systematic and site-dependent deviations in high- and low-frequency power and the LF/HF ratio (49). Estimates derived from photoplethysmography should therefore not be treated as directly equivalent to electrocardiographic HRV. No study used 24-hour ambulatory HRV, and long-term retention beyond 4 weeks of detraining is uncharacterized. No single study included both an acute and a chronic arm with identical recording protocols, constraining the acute–chronic comparison. A final limitation concerns generalizability: the higher-risk but non-clinical populations that motivate a preventive application of IMT, including shift workers, emergency responders, and adults exposed to neurotoxic or cardiotropic insults, have not themselves been studied, so the present evidence is extrapolated from broadly healthy volunteers and requires confirmation in those target groups.

These gaps map directly onto priorities for future work. A sex-balanced randomized trial in mid-life adults is needed, for example 50% MIP over 8 weeks with a pre-specified sex-by-time interaction. Ambulatory 24-hour HRV recording would help capture circadian autonomic dynamics. A formal multi-arm dose–response design, for example 30%, 50% and 70% MIP against sham, is needed to test for differences between intensity strata. Post-cessation recordings at 4, 8 and 12 weeks would help establish whether any adaptation is retained. Standardized reporting of a minimum HRV dataset would substantially improve the comparability of future trials.

## 5 Conclusion

Reduced heart rate variability is among the earliest measurable signs of cardiac autonomic decline and has been associated with subsequent cardiovascular morbidity. In the healthy adult samples studied to date, inspiratory muscle training was associated with changes in HRV in a potentially favourable direction, although these changes may partly reflect altered breathing mechanics rather than autonomic adaptation. Low-to-moderate-intensity loading (30%–60% MIP) was associated with increases in vagal HRV indices, detectable after a single session and as chronic adaptations from the second week, whereas sustained or fatiguing loading above approximately 60% MIP was associated with a relative shift toward higher low-frequency power, consistent with metaboreflex activation. The certainty of this evidence is very low. Included reports were few and small, largely originated from three research groups, and were confined to older women and young trained men. A protocol of 50% MIP, 30 breaths per session, five sessions per week for at least four weeks, with HRV recorded under standardized resting conditions, is proposed as a reference condition for future trials. IMT is inexpensive, requires minimal facilities, and can be self-administered, making it practical to evaluate in groups with elevated autonomic risk with poor adherence to conventional exercise, including shift workers, emergency responders, and adults exposed to neurotoxic or cardiotropic insults. None of these populations has been studied to date. Adequately powered, sex-balanced trials in mid-life adults, multi-arm intensity comparisons, replication beyond the current geographical concentrations, and post-training follow-up are priorities to determine whether these autonomic changes are durable and clinically meaningful. Independent, multicentre and geographically diverse studies are required before any clinical or occupational preventive recommendation can be considered.

## Data Availability

The original contributions presented in the study are included in the article/Supplementary Material, further inquiries can be directed to the corresponding author.

## References

[1] Malik M. Heart rate variability: standards of measurement, physiological interpretation, and clinical use. Circulation. (1996) 93(5):1043–65. 10.1161/01.cir.93.5.1043 8598068

[2] Wang BX Brennand E L Page P Mitchell ARJ. Heart rate variability in cardiovascular disease diagnosis, prognosis and management. Front Cardiovasc Med. (2026) 12:1680783. 10.3389/fcvm.2025.1680783 41669240 PMC12883741

[3] Shaffer F Ginsberg JP. An overview of heart rate variability metrics and norms. Front Public Health. (2017) 5:258. 10.3389/fpubh.2017.00258 29034226 PMC5624990

[4] Sassi R Cerutti S Lombardi F Malik M Huikuri HV Peng C-K. Advances in heart rate variability signal analysis: joint position statement by the e-cardiology ESC working group and the European heart rhythm association. Europace. (2015) 17(9):1341–53. 10.1093/europace/euv015 26177817

[5] Mello PR Guerra GM Borile S Rondon MU Alves MJ Negrão CE. Inspiratory muscle training reduces sympathetic nervous activity and improves inspiratory muscle weakness and quality of life in patients with chronic heart failure: a clinical trial. J Cardiopulm Rehabil Prev. (2012) 32(5):255–61. 10.1097/HCR.0b013e31825828da 22785143

[6] McConnell A Gosselink R Hogarth B. Respiratory Muscle Training: Theory and Practice. 1st ed. Edinburgh: Elsevier/Churchill Livingstone (2013).

[7] Craighead DH Heinbockel TC Freeberg KA Rossman MJ Jackman RA Jankowski LR. Time-efficient inspiratory muscle strength training lowers blood pressure and improves endothelial function, NO bioavailability, and oxidative stress in midlife/older adults with above-normal blood pressure. J Am Heart Assoc. (2021) 10(13):e020980. 10.1161/JAHA.121.020980 34184544 PMC8403283

[8] Freeberg KA Craighead DH Heinbockel TC. Time-efficient, high-resistance inspiratory muscle strength training increases cerebrovascular reactivity in midlife and older adults. Am J Physiol Heart Circ Physiol. (2023) 325(5):H1059–68. 10.1152/ajpheart.00385.2023 37682232 PMC10908405

[9] Zheng SQ Zhang Q Li SY Li S Yao Q Zheng X. Effects of inspiratory muscle training in patients with hypertension: a meta-analysis. Front Cardiovasc Med. (2023) 10:1113509. 10.3389/fcvm.2023.1113509 37332584 PMC10270119

[10] Fan K Cao W Chang H Tian F. A hypotensive protocol of inspiratory muscle strength training: systematic review and meta-analysis with trial sequential analysis. J Clin Hypertens (Greenwich). (2023) 25(11):971–82. 10.1111/jch.14732 37803506 PMC10631095

[11] Ferreira JB Plentz RD Stein C Casali KR Arena R Lago PD. Inspiratory muscle training reduces blood pressure and sympathetic activity in hypertensive patients: a randomized controlled trial. Int J Cardiol. (2013) 166(1):61–7. 10.1016/j.ijcard.2011.09.069 21985749

[12] De Abreu RM Rehder-Santos P Minatel V D Santos GL Catai AM. Effects of inspiratory muscle training on cardiovascular autonomic control: a systematic review. Auton Neurosci. (2017) 208:29–35. 10.1016/j.autneu.2017.09.002 28916152

[13] Cipriano GFB Cipriano Jr. G Santos FV Güntzel Chiappa AM Pires L Cahalin LP. Current insights of inspiratory muscle training on the cardiovascular system: a systematic review with meta-analysis. Integr Blood Press Control. (2019) 12:1–11. 10.2147/IBPC.S159386 31190975 PMC6535083

[14] Manifield J Winnard A Hume E Armstrong M Baker K Adams N. Inspiratory muscle training for improving inspiratory muscle strength and functional capacity in older adults: a systematic review and meta-analysis. Age Ageing. (2021) 50(3):716–24. 10.1093/ageing/afaa221 33951159

[15] Bernardi L Porta C Spicuzza L Bellwon J Spadacini G Frey AW. Slow breathing increases arterial baroreflex sensitivity in patients with chronic heart failure. Circulation. (2002) 105(2):143–5. 10.1161/hc0202.103311 11790690

[16] Hayano J Yuda E. Pitfalls of assessment of autonomic function by heart rate variability. J Physiol Anthropol. (2019) 38(1):3. 10.1186/s40101-019-0193-2 30867063 PMC6416928

[17] DeLucia CM, DeBonis DR, Schwyhart SM, Bailey EF. Acute cardiovascular responses to a single bout of high intensity inspiratory muscle strength training in healthy young adults. J Appl Physiol (1985). (2021) 130(4):1114–21. 10.1152/japplphysiol.01015.2020 33600284

[18] St Croix CM Morgan BJ Wetter TJ Dempsey JA. Fatiguing inspiratory muscle work causes reflex sympathetic activation in humans. J Physiol. (2000) 529(Pt 2):493–504. 10.1111/j.1469-7793.2000.00493.x 11101657 PMC2270191

[19] Witt JD Guenette JA Rupert JL McKenzie DC Sheel AW. Inspiratory muscle training attenuates the human respiratory muscle metaboreflex. J Physiol. (2007) 584(Pt 3):1019–28. 10.1113/jphysiol.2007.140855 17855758 PMC2277000

[20] Smith JR Joyner MJ Curry TB. Consequences of group III/IV afferent feedback and respiratory muscle work on exercise tolerance in heart failure with reduced ejection fraction. Exp Physiol. (2023) 108(10):1351–63. 10.1113/EP091210 37735814 PMC10900130

[21] Rodrigues GD Gurgel JL Da Nóbrega ACL Soares PP. Inspiratory muscle training improves breathing pattern and sympathovagal balance but not spontaneous baroreflex sensitivity in older women. Respir Physiol Neurobiol. (2021) 291:103685. 10.1016/j.resp.2021.103672 33866039

[22] Monaghan AS Johansson H Torres A Brewer GA Peterson DS. Respiratory training in older women: unravelling central and peripheral hemodynamic slow oscillatory patterns. Exp Gerontol. (2022) 162:111759. 10.1016/j.exger.2022.111759 36529363

[23] Erdem JS Das MK De Ryck E Skare Ø Lie J-AS Bugge M. Night shift work and indicators of cardiovascular risk: a systematic review and meta-analysis. Environ Res. (2025) 276:121503. 10.1016/j.envres.2025.121503 40164421

[24] Teixeira IG Verzola MR Filipini RE Speretta GF. The effects of a firefighting simulation on the vascular and autonomic functions and cognitive performance: a randomized crossover study. Front Physiol. (2023) 14:1215006. 10.3389/fphys.2023.1215006 37811491 PMC10551144

[25] B Stella A Ajčević M Furlanis G. Cardiovascular autonomic dysfunction in “long COVID”: pathophysiology, heart rate variability, and inflammatory markers. Front Cardiovasc Med. (2023) 10:1256512. 10.3389/fcvm.2023.1256512 37719983 PMC10502909

[26] Yoon SY Oh J. Cardiovascular autonomic dysfunction before and after chemotherapy in cancer patients. J Clin Neurol. (2024) 20(6):551–62. 10.3988/jcn.2024.0221 39505307 PMC11543394

[27] Page MJ McKenzie JE Bossuyt PM Boutron I Hoffmann TC Mulrow CD. The PRISMA 2020 statement: an updated guideline for reporting systematic reviews. Br Med J. (2021) 372:n71. 10.1136/bmj.n71 PMC8005924 33782057

[28] Sterne JAC Savović J Page MJ Elbers RG Blencowe NS Boutron I. Rob 2: a revised tool for assessing risk of bias in randomised trials. Br Med J. (2019) 366:l4898. 10.1136/bmj.l4898 31462531

[29] Sterne JAC Hernán MA Reeves BC Savović J Berkman ND Viswanathan M. ROBINS-I: a tool for assessing risk of bias in non-randomised studies of interventions. Br Med J. (2016) 355:i4919. 10.1136/bmj.i4919 PMC5062054 27733354

[30] Tarvainen MP Niskanen JP Lipponen JA Ranta-Aho PO Karjalainen PA. Kubios HRV: heart rate variability analysis software. Comput Methods Programs Biomed. (2014) 113(1):210–20. 10.1016/j.cmpb.2013.07.024 24054542

[31] Martins de Abreu R Porta A Rehder-Santos P Cairo B Donisete da Silva C De Favari Signini É. Effects of inspiratory muscle training intensity on cardiovascular control in amateur cyclists. Am J Physiol Regul Integr Comp Physiol. (2019) 317(5):R891–902. 10.1152/ajpregu.00167.2019 31596110

[32] De Abreu RM Catai AM Cairo B Rehder-Santos P Da Silva CD Signini ÉF. A transfer entropy approach for the assessment of the impact of inspiratory muscle training on the cardiorespiratory coupling of amateur cyclists. Front Physiol. (2020) 11:134. 10.3389/fphys.2020.00134 32158402 PMC7052290

[33] Rodrigues GD Dal Lago P Da Silva Soares PP. Time-dependent effects of inspiratory muscle training and detraining on cardiac autonomic control in older women. Exp Gerontol. (2021) 150:111357. 10.1016/j.exger.2021.111357 33864832

[34] Archiza B Andaku DK Beltrame T Borghi-Silva A Catai AM. Acute effects of different inspiratory resistive loading on heart rate variability in healthy elderly patients. Braz J Phys Ther. (2013) 17(4):401–8. 10.1590/S1413-35552012005000104 23970114

[35] Rodrigues GD Gurgel JL Gonçalves TR Soares PPS. Inspiratory muscle training improves physical performance and cardiac autonomic modulation in older women. Eur J Appl Physiol. (2018) 118(6):1143–52. 10.1007/s00421-018-3844-9 29549494

[36] Frazão M Santos AC Araújo AA Romualdo MP De Mello BLC Jerônimo GG. Acute effects of inspiratory loading in older women: where the breath meets the heart. Respir Physiol Neurobiol. (2021) 290:103673. 10.1016/j.resp.2021.103673

[37] Ladriñán-Maestro A Sánchez-Infante J Martín-Vera D Sánchez-Sierra A. Influence of an inspiratory muscle fatigue protocol on healthy youths on respiratory muscle strength and heart rate variability: a randomized controlled trial. Front Physiol. (2023) 14:1257656. 10.3389/fphys.2023.1257656 PMC11377326 39247158

[38] Ladriñán-Maestro A Sánchez-Infante J Martín-Vera D Del-Blanco-Muñiz JA Merino-Andrés J Sánchez-Sierra A. Influence of an inspiratory muscle fatigue protocol on older adults on respiratory muscle strength and heart rate variability: a randomized controlled trial. Front Neurosci. (2024) 18:1423927. 10.3389/fnins.2024.1423927 PMC11588671 39600654

[39] Tanrıverdi A Savci S Kahraman BO. Acute effects of inspiratory muscle training at different intensities in healthy young people. Ir J Med Sci. (2021) 190(2):577–85. 10.1007/s11845-020-02355-8 32851483

[40] Souza HCD Roscani MG Veiga GL. Effect of different intensities of inspiratory muscle exercise on cardiac autonomic control in the post-exercise recovery in healthy adults. Fisioter Pesqui. (2024) 31:e23023324. 10.1590/1809-2950/e23023324en

[41] Hayano J Yuda E. Assessment of autonomic function by long-term heart rate variability: beyond the classical framework of LF and HF measurements. J Physiol Anthropol. (2021) 40(1):21. 10.1186/s40101-021-00272-y 34847967 PMC8630879

[42] Kaminski DM Schaan BD Da Silva AMV Soares PP Dall’Ago P. Inspiratory muscle training in patients with diabetic autonomic neuropathy: a randomized clinical trial. Clin Auton Res. (2015) 25(4):263–6. 10.1007/s10286-015-0291-0 25982993

[43] Cutrim ALC Duarte AAM Silva-Filho AC Dias CJ Urtado CB Ribeiro RM. Inspiratory muscle training improves autonomic modulation and exercise tolerance in chronic obstructive pulmonary disease subjects: a randomized-controlled trial. Respir Physiol Neurobiol. (2019) 263:31–7. 10.1016/j.resp.2019.03.003 30853602

[44] Huang C-C Lai Y-R Wu F-A Kuo N-Y Tsai Y-C Cheng B-C. Simultaneously improved pulmonary and cardiovascular autonomic function and short-term functional outcomes in patients with Parkinson’s disease after respiratory muscle training. J Clin Med. (2020) 9(2):316. 10.3390/jcm9020316 31979103 PMC7074532

[45] Craighead DH Freeberg KA McCarty NP Seals DR. Time-efficient, high-resistance inspiratory muscle strength training for cardiovascular aging. Exp Gerontol. (2021) 154:111515. 10.1016/j.exger.2021.111515 34389471 PMC9150656

[46] Caicedo-Trujillo S Torres-Castro R Vasconcello-Castillo L. The effectiveness of respiratory muscular training in athletes: a systematic review and meta-analysis. Sci Sports. (2025) 40(2):105–14. 10.1016/j.jbmt.2025.01.010

[47] Flahr H Brown WJ Kolbe-Alexander TL. A systematic review of physical activity-based interventions in shift workers. Prev Med Rep. (2018) 10:323–31. 10.1016/j.pmedr.2018.04.004 29868387 PMC5984233

[48] Bourdillon N Schmitt L Yazdani S Vesin JM Millet GP. Minimal window duration for accurate HRV recording in athletes. Front Neurosci. (2017) 11:456. 10.3389/fnins.2017.00456 28848382 PMC5554345

[49] Yuda E Shibata M Ogata Y Ueda N Yambe T Yoshizawa M. Pulse rate variability: a new biomarker, not a surrogate for heart rate variability. J Physiol Anthropol. (2020) 39(1):21. 10.1186/s40101-020-00233-x 32811571 PMC7437069

