# Supplementary material for: Inspiratory muscle training and cardiac autonomic function in healthy adults: a systematic review of acute and chronic responses
Source: Front Cardiovasc Med. 2026 Sep 4;13:1935324. doi: 10.3389/fcvm.2026.1935324 (PMC13585652; doi:10.3389/fcvm.2026.1935324)
Supplement: Supplementary file 1 [file Datasheet1.pdf]

Supplementary Material

The following materials are provided as separate supplementary files and are not part of the main manuscript

Supplementary File S1. Complete database-specific search strategies (PubMed, Scopus, Web of Science, MEDLINE, EMBASE, Dimensions), including controlled-vocabulary and free-text terms, field tags, Boolean syntax, and the date of the last search.

Supplementary Table S2. Risk-of-bias assessment of the single non-randomised study (DeLucia 2021) using ROBINS-I.

Figure legends

Figure 1. PRISMA 2020 flow diagram of study identification, screening, eligibility assessment, and inclusion.

Figure 2. Risk-of-bias traffic-light plot (RoB 2 for randomised studies across domains and overall, by study, using a colour-blind-safe palette.

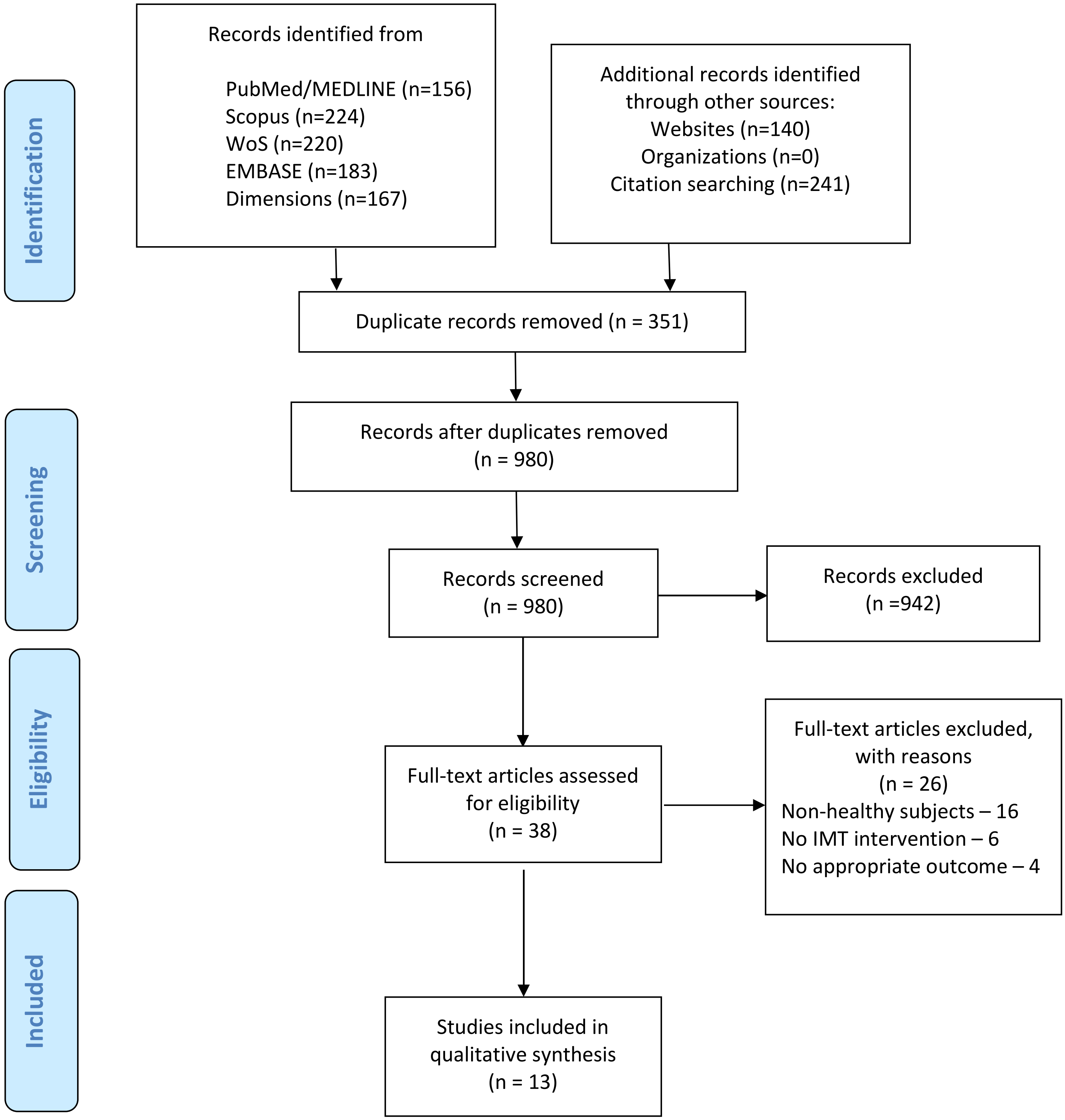

Figure 1. PRISMA flow diagram showing study selection process from initial database selections through final inclusion

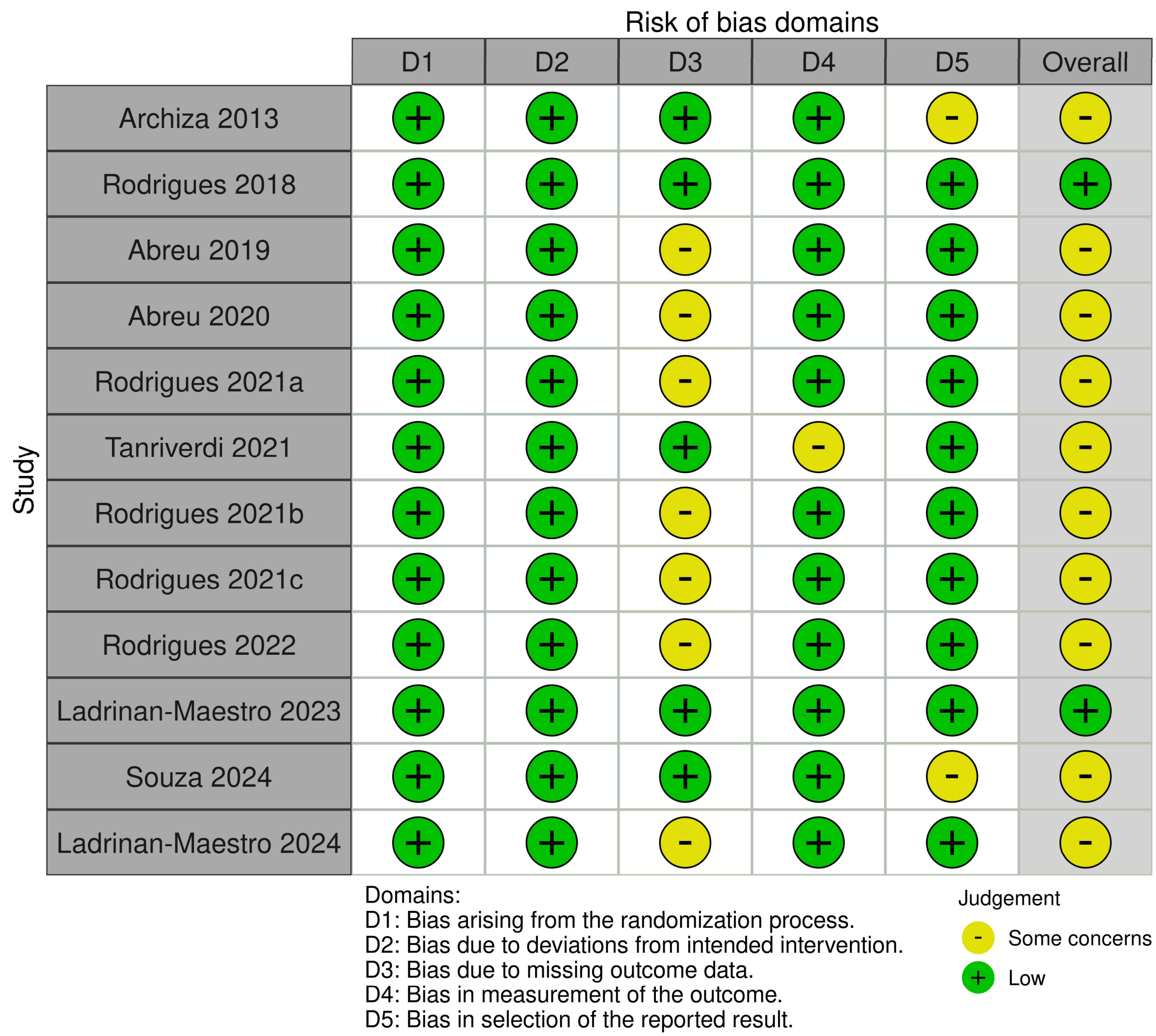

Figure 2: Risk-of-bias traffic-light plot (RoB 2) for the twelve randomised reports, showing domain-level and overall judgements by study, using a colour-blind-safe palette.

Supplementary File 1. Search Strategy and Study Selection

Electronic Database Search Strategy
Databases Searched: PubMed, Scopus, Web of Science, MEDLINE, EMBASE, Dimensions
Time Frame: Inception to 23rd April 2026

PubMed/MEDLINE Search Strategy

("Inspiratory Muscle Training"[Mesh] OR "inspiratory muscle training"[Title/Abstract] OR "inspiratory muscle strength training"[Title/Abstract] OR "inspiratory muscle strength exercise"[Title/Abstract] OR "inspiratory muscle exercise"[Title/Abstract] OR "respiratory muscle training"[Title/Abstract] OR "respiratory muscle strengthening"[Title/Abstract] OR "respiratory muscle exercise"[Title/Abstract] OR "breathing muscle training"[Title/Abstract] OR "breathing muscle strengthening"[Title/Abstract] OR "loaded breathing"[Title/Abstract] OR "inspiratory loading"[Title/Abstract] OR "pressure threshold loading"[Title/Abstract] OR "threshold inspiratory muscle training"[Title/Abstract] OR "threshold loading"[Title/Abstract] OR IMT[Title/Abstract] OR IMST[Title/Abstract] OR "respiratory training"[Title/Abstract]) AND ("Heart Rate Variability"[Mesh] OR "heart rate variability"[Title/Abstract] OR HRV[Title/Abstract] OR "cardiac autonomic function"[Title/Abstract] OR "cardiac autonomic control"[Title/Abstract] OR "autonomic function"[Title/Abstract] OR "autonomic nervous system"[Title/Abstract] OR "autonomic modulation"[Title/Abstract] OR "autonomic regulation"[Title/Abstract] OR "sympathetic modulation"[Title/Abstract] OR "parasympathetic modulation"[Title/Abstract] OR "vagal modulation"[Title/Abstract] OR "sympathovagal balance"[Title/Abstract] OR "sympathetic-vagal balance"[Title/Abstract] OR "vagal tone"[Title/Abstract] OR "vagal activity"[Title/Abstract] OR "cardiac vagal control"[Title/Abstract] OR "muscle sympathetic nerve activity"[Title/Abstract] OR MSNA[Title/Abstract] OR "sympathetic nerve activity"[Title/Abstract] OR "baroreflex sensitivity"[Title/Abstract] OR "cardiorespiratory coupling"[Title/Abstract] OR "respiratory sinus arrhythmia"[Title/Abstract] OR RSA[Title/Abstract] OR RMSSD[Title/Abstract] OR SDNN[Title/Abstract] OR pNN50[Title/Abstract] OR "high frequency power"[Title/Abstract] OR HF[Title/Abstract] OR "low frequency power"[Title/Abstract] OR LF[Title/Abstract] OR "LF/HF ratio"[Title/Abstract] OR "sample entropy"[Title/Abstract] OR SD1[Title/Abstract] OR SD2[Title/Abstract]) AND("Blood Pressure"[Mesh] OR "blood pressure"[Title/Abstract] OR "systolic blood pressure"[Title/Abstract] OR "diastolic blood pressure"[Title/Abstract] OR "resting blood pressure"[Title/Abstract] OR hypertension[Title/Abstract] OR normotension[Title/Abstract] OR"resting heart rate"[Title/Abstract] OR "heart rate"[Title/Abstract] OR cardiovascular[Title/Abstract] OR "cardiovascular outcomes"[Title/Abstract] OR"vascular function"[Title/Abstract] OR endothelial[Title/Abstract] OR"arterial stiffness"[Title/Abstract] OR "pulse wave velocity"[Title/Abstract] OR "aortic pulse wave velocity"[Title/Abstract] OR"cerebrovascular reactivity"[Title/Abstract])

Humans; English language

Scopus Search Strategy

TITLE-ABS-KEY (("inspiratory muscle training" OR "inspiratory muscle strength training" OR "inspiratory muscle strength exercise" OR "inspiratory muscle exercise" OR "respiratory muscle training" OR "respiratory muscle strengthening" OR "respiratory muscle exercise" OR "breathing muscle training" OR "breathing muscle strengthening" OR "loaded breathing" OR "inspiratory loading" OR "pressure threshold loading" OR "threshold inspiratory muscle training" OR "threshold loading" OR IMT OR IMST OR "respiratory training") AND ("heart rate variability" OR HRV OR "cardiac autonomic function" OR "cardiac autonomic control" OR "autonomic function" OR "autonomic nervous system" OR "autonomic modulation" OR "autonomic regulation" OR "sympathetic

modulation" OR "parasympathetic modulation" OR "vagal modulation" OR "sympathovagal balance" OR "sympathetic-vagal balance" OR "vagal tone" OR "vagal activity" OR "cardiac vagal control" OR "muscle sympathetic nerve activity" OR MSNA OR "sympathetic nerve activity" OR "baroreflex sensitivity" OR "cardiorespiratory coupling" OR "respiratory sinus arrhythmia" OR RSA OR RMSSD OR SDNN OR pNN50 OR "high frequency power" OR HF OR "low frequency power" OR LF OR "LF/HF ratio" OR "sample entropy" OR SD1 OR SD2) AND ("blood pressure" OR "systolic blood pressure" OR "diastolic blood pressure" OR" resting blood pressure" OR hypertension OR normotension OR" resting heart rate" OR" heart rate" OR cardiovascular OR "cardiovascular outcomes" OR" vascular function" OR endothelial OR "arterial stiffness" OR "pulse wave velocity" OR "aortic pulse wave velocity" OR "cerebrovascular reactivity"))

Language: English

Web of Science Search Strategy

TS= (("inspiratory muscle training" OR "inspiratory muscle strength training" OR "inspiratory muscle strength exercise" OR "inspiratory muscle exercise" OR "respiratory muscle training" OR "respiratory muscle strengthening" OR "respiratory muscle exercise" OR "breathing muscle training" OR "breathing muscle strengthening" OR "loaded breathing" OR "inspiratory loading" OR "pressure threshold loading" OR "threshold inspiratory muscle training" OR "threshold loading" OR IMT OR IMST OR "respiratory training") AND ("heart rate variability" OR HRV OR "cardiac autonomic function" OR "cardiac autonomic control" OR "autonomic function" OR "autonomic nervous system" OR "autonomic modulation" OR "autonomic regulation" OR "sympathetic modulation" OR "parasympathetic modulation" OR "vagal modulation" OR "sympathovagal balance" OR "sympathetic-vagal balance" OR "vagal tone" OR "vagal activity" OR "cardiac vagal control" OR "muscle sympathetic nerve activity" OR MSNA OR "sympathetic nerve activity" OR "baroreflex sensitivity" OR "cardiorespiratory coupling" OR "respiratory sinus arrhythmia" OR RSA OR RMSSD OR SDNN OR pNN50 OR "high frequency power" OR HF OR "low frequency power" OR LF OR "LF/HF ratio" OR "sample entropy" OR SD1 OR SD2) AND ("blood pressure" OR "systolic blood pressure" OR "diastolic blood pressure" OR "resting blood pressure" OR hypertension OR normotension OR "resting heart rate" OR "heart rate" OR cardiovascular OR "cardiovascular outcomes" OR "vascular function" OR endothelial OR "arterial stiffness" OR "pulse wave velocity" OR "aortic pulse wave velocity" OR "cerebrovascular reactivity")))

Languages: English

EMBASE Search Strategy

('inspiratory muscle training'/exp OR 'inspiratory muscle training':ti,ab OR 'inspiratory muscle strength training':ti,ab OR 'respiratory muscle training':ti,ab OR 'respiratory muscle strengthening':ti,ab OR 'loaded breathing':ti,ab OR 'inspiratory loading':ti,ab OR 'threshold inspiratory muscle training':ti,ab OR IMT:ti,ab OR IMST:ti,ab) AND ('heart rate variability'/exp OR 'heart rate variability':ti,ab OR HRV:ti,ab OR 'autonomic nervous system'/exp OR 'autonomic function':ti,ab OR 'cardiac autonomic control':ti,ab OR 'baroreflex sensitivity':ti,ab OR 'respiratory sinus arrhythmia':ti,ab OR RMSSD:ti,ab OR SDNN:ti,ab OR pNN50:ti,ab OR HF:ti,ab OR LF:ti,ab OR 'LF/HF ratio':ti,ab OR SD1:ti,ab OR SD2:ti,ab) AND ('blood pressure'/exp OR 'blood pressure':ti,ab OR 'systolic blood pressure':ti,ab OR 'diastolic blood pressure':ti,ab OR hypertension:ti,ab OR 'heart rate':ti,ab OR 'vascular function':ti,ab OR 'arterial stiffness':ti,ab OR 'pulse wave velocity':ti,ab OR 'cerebrovascular reactivity':ti,ab)

AND [humans]/lim AND [english]/lim

Dimensions Search Strategy

(("inspiratory muscle training" OR "inspiratory muscle strength training" OR "inspiratory muscle strength exercise" OR "inspiratory muscle exercise" OR "respiratory muscle training" OR "respiratory muscle strengthening" OR "respiratory muscle exercise" OR "breathing muscle training" OR "breathing muscle strengthening" OR "loaded breathing" OR "inspiratory loading" OR "pressure threshold loading" OR "threshold inspiratory muscle training" OR "threshold loading" OR IMT OR IMST OR "respiratory training") AND ("heart rate variability" OR HRV OR "cardiac autonomic function" OR "cardiac autonomic control" OR "autonomic function" OR "autonomic nervous system" OR "autonomic modulation" OR "autonomic regulation" OR "sympathetic modulation" OR "parasympathetic modulation" OR "vagal modulation" OR "sympathovagal balance" OR "sympathetic-vagal balance" OR "vagal tone" OR "vagal activity" OR "cardiac vagal control" OR "muscle sympathetic nerve activity" OR MSNA OR "sympathetic nerve activity" OR "baroreflex sensitivity" OR "cardiorespiratory coupling" OR "respiratory sinus arrhythmia" OR RSA OR RMSSD OR SDNN OR pNN50 OR "high frequency power" OR HF OR "low frequency power" OR LF OR "LF/HF ratio" OR "sample entropy" OR SD1 OR SD2) AND ("blood pressure" OR "systolic blood pressure" OR "diastolic blood pressure" OR" resting blood pressure" OR hypertension OR normotension OR" resting heart rate" OR" heart rate" OR cardiovascular OR "cardiovascular outcomes" OR" vascular function" OR endothelial OR "arterial stiffness" OR "pulse wave velocity" OR "aortic pulse wave velocity" OR "cerebrovascular reactivity"))

AND English AND Article

Supplementary Table S2. Risk-of-bias assessment of the single non-randomised study (ROBINS-I).

Domain	Judgement	Rationale
D1. Confounding	Serious	Single-arm design with no comparator or sham. Time-related, postural and procedural effects cannot be separated from the effect of the load.
D2. Selection of participants	Low	Prospective recruitment; start of intervention coincided with start of follow-up.
D3. Classification of interventions	Low	Intervention defined a priori (5 × 6 breaths at 75% PImax) and verified by pneumotachometer.
D4. Deviations from intended interventions	Low	Single supervised session with standardised cueing and breathing rate; no deviations reported.
D5. Missing data	Moderate	Complete datasets except one participant in whom MSNA could not be recorded; no sensitivity analysis reported.
D6. Measurement of outcomes	Moderate	Microneurography is a reference method, but assessors could not be blinded and burst identification involves operator judgement.
D7. Selection of the reported result	Moderate	No prospective registration or published protocol identified.
**Overall**	**Serious**	Driven by confounding; without a comparator, observed changes cannot be attributed to inspiratory loading.

DeLucia 2021 [17] was the only non-randomised, single-arm before–after study and was assessed with ROBINS-I. The other 11 randomised reports were assessed with RoB 2 and shown in Figure 2. ROBINS-I categories: low, moderate, serious, critical, no information.
